# Marine-Derived *Enterococcus faecalis* HY0110 as a Next-Generation Functional Food Probiotic: Comprehensive In Vitro and In Vivo Bioactivity Evaluation and Synergistic Fermentation of *Periplaneta americana* Extract Powder

**DOI:** 10.3390/foods14071181

**Published:** 2025-03-28

**Authors:** Feiyun Huang, Nan Yang, Qingqing Zhang, Cuiling Luo, Jingheng Wang, Yu Yang, Bisong Yue, Peng Chen, Xiuyue Zhang

**Affiliations:** 1 Key Laboratory of Bio-Resources and Eco-Environment (Ministry of Education), College of Life Sciences, Sichuan University, Chengdu 610065, China; fyunhuang@163.com (F.H.); zqingqing0822@163.com (Q.Z.); luocuiling2019@163.com (C.L.); wangjingheng@stu.scu.edu.cn (J.W.); yy20270630@163.com (Y.Y.); bsyue@scu.edu.cn (B.Y.); 2 Key Laboratory of Qinghai-Tibetan Plateau Animal Genetic Resource Reservation and Utilization, Sichuan Province and Ministry of Education, Southwest Minzu University, Chengdu 610041, China; yangnan0204@126.com; 3 MOE Key Laboratory of Deep Earth Science and Engineering, College of Architecture and Environment, Sichuan University, Chengdu 610065, China; 4 Sichuan Key Laboratory of Conservation Biology on Endangered Wildlife, College of Life Sciences, Sichuan University, Chengdu 610065, China

**Keywords:** functional food, marine-derived probiotic, *Enterococcus faecalis*, insect-derived ingredients, fermentation biotechnology, bioactive metabolites

## Abstract

Addressing the escalating global burdens of inflammatory bowel disease and antimicrobial resistance demanded innovative food-based approaches to fortify gut health and suppress pathogens. We introduced a novel edible probiotic, *Enterococcus faecalis* HY0110, isolated from marine *Thunnus thynnus*. Through comprehensive in vitro, in vivo, and metabolomic analyses, we demonstrated its superior antibacterial effects compared to *Lactobacillus rhamnosus* GG, along with significantly enhanced antioxidant and free-radical scavenging capacities. Notably, elevated acetic acid production strongly correlated with its antimicrobial efficacy (R ≥ 0.999). HY0110 also exerted antiproliferative effects on HT-29 colorectal cancer cells by attenuating β-catenin and BCL-2 expression while upregulating pro-apoptotic markers P62 and c-PARP. In a DSS-induced colitis model, HY0110 alleviated inflammation, restored gut microbial homeostasis, and enhanced deterministic processes in community assembly dynamics. Furthermore, fermenting *Periplaneta americana* powder with HY0110 triggered extensive metabolic remodeling, notably a 668.73-fold rise in astragaloside A, plus increases in L-Leucyl-L-Alanine, S-lactoylglutathione, and 16,16-dimethyl prostaglandin A1. These shifts diminished harmful components and amplified essential amino acids and peptides to bolster immune modulation, redox balance, and anti-inflammatory responses. This work established a transformative paradigm for utilizing marine probiotics and novel entomological substrates in functional foods, presenting strategic pathways for precision nutrition and inflammatory disease management.

## 1. Introduction

Global challenges—including the rapid rise of inflammatory bowel disease (IBD), escalating antibiotic resistance, and the growing prevalence of colorectal cancer—underscore the urgent need for novel interventions beyond conventional medical therapies [1]. While pharmacological solutions remain indispensable, the food science community increasingly recognizes the pivotal role of diet and functional foods in preserving gut microbial equilibrium and overall health. In particular, the widespread overuse of antibiotics—across both clinical and agricultural sectors—has heightened fears of drug-resistant pathogens, intensifying the search for safe natural alternatives [2]. In response, probiotic-based strategies have gained considerable traction for their ability to maintain gut homeostasis, attenuate inflammatory processes, and potentially diminish reliance on conventional antibiotics [3,4,5].

Within the spectrum of probiotics investigated for these purposes, *Enterococcus faecalis* has attracted considerable attention for its adaptability and multifunctional capacity. As a facultatively anaerobic Gram-positive bacterium, it colonizes the gastrointestinal tract with relative ease and produces various antimicrobial substances, including organic acids, hydrogen peroxide, and bacteriocins such as *EF478*. These compounds lower local pH and compromise pathogenic bacterial membranes, thereby contributing to both food safety and a balanced gut microbiota [6]. Additionally, *E. faecalis* appears to modulate immune pathways, likely through mechanisms that suppress excessive inflammatory responses [7]. Compelling preliminary data also indicate that its bacteriocins may selectively inhibit tumor cells while sparing normal tissues, suggesting potential antitumor efficacy [8].

Despite these promising attributes, certain strains of *E. faecalis* possess virulence factors (e.g., *asa1*, *efaA*, and *ace*) and antibiotic resistance genes (e.g., *optrA*, *fexA*), highlighting the need for meticulous strain-specific safety assessments [9]. Moreover, notable functional heterogeneity within the same species underscores the importance of rigorous screening protocols to identify strains offering consistent multifaceted benefits while minimizing risks. Traditional single-parameter evaluations (e.g., focusing solely on antibacterial properties) often overlook the intricate in vivo interactions pivotal to immune modulation and broader ecological impacts. Recent advances in multi-omics methodologies (e.g., genomics, transcriptomics, proteomics, and metabolomics)—which enable the comprehensive analysis of biological systems—and AI-driven high-throughput screening—which facilitates the rapid and efficient evaluation of large sample sets using artificial intelligence—support the adoption of integrated assessment frameworks [10,11,12].

Extreme and specialized environments represent promising reservoirs for discovering novel probiotics. The marine ecosystem, for example, imposes strong selective pressures that can endow microbial residents with exceptional stress tolerance and metabolic versatility [13]. The gut microbiota of marine fish, such as tuna (*Thunnus thynnus* and *Thunnus obesus*), is adapted to high-pressure, low-temperature, and osmotically dynamic conditions, often exhibiting unique functional traits [14]. Investigating *E. faecalis* isolates from these habitats could, therefore, unveil novel strains with enhanced resilience and health benefits.

In tandem with these probiotic advances, emerging functional ingredients are also drawing attention for their capacity to improve the nutritional and bioactive profile of human diets. Insect-derived products, notably *Periplaneta americana*, have piqued interest due to their high protein content, complete essential amino acid profiles (e.g., lysine, methionine, and tryptophan), immunomodulatory peptides (defensins and cecropins), and environmental sustainability with substantially lower ecological footprints compared to conventional protein sources [15]. Nonetheless, concerns regarding bioavailability and stability pose barriers to broader adoption. Probiotic fermentation strategies offer a viable solution by breaking down complex molecules and generating bioactive metabolites [16]. When judiciously combined, such novel functional ingredients and probiotics can deliver synergistic advantages: strengthening gut health through prebiotic–probiotic interactions, modulating immune responses via complementary signaling pathways, and enhancing overall well-being through improved nutrient absorption and metabolic regulation.

Against this backdrop, the present study investigates a tuna-derived *E. faecalis* strain, HY0110, for its multifunctional benefits in the realm of functional foods. Specifically, we aim to (i) develop a comprehensive assessment framework encompassing antibacterial activity against clinically relevant pathogens, antioxidant capacity through multiple radical scavenging assays, and immunomodulatory evaluations via cytokine profiling to capture the strain’s broad-spectrum activities; (ii) examine its in vivo efficacy by incorporating HY0110 into food formulations and monitoring shifts in metabolic indices, gut microbiota composition, and inflammatory markers; and (iii) explore metabolomic alterations in *P. americana*-based substrates following fermentation with HY0110, thereby enhancing their nutritional and bioactive profiles. By adopting this integrated approach, we seek to advance the scientific basis for probiotic-driven functional foods and provide actionable insights for improving product quality, public health, and environmental sustainability in an era of increasing antimicrobial resistance and inflammatory disorders.

## 2. Materials and Methods

### 2.1. Experimental Strains

The *E. faecalis* strain HY0110, preserved in our laboratory culture collection, was originally isolated from the gastrointestinal tract of *T. thynnus* using calcium carbonate-containing MRS agar plates. Bacterial 16S rRNA gene identification was performed with universal primers 27F (5′-AGAGTTTGATCCTGGCTCAG-3′) and 1492R (5′-GGTTACCTTGTTACGACTT-3′). PCR amplification was carried out in a 25 μL reaction mixture containing 1 μL of template DNA, 1 μL of each primer, 12.5 μL of Taq MasterMix, and 9.5 μL of ddH_2_O. The thermal cycling protocol consisted of an initial denaturation at 95 °C for 5 min; 30 cycles of 95 °C for 30 s, 54 °C for 30 s, 72 °C for 45 s; and a final extension at 72 °C for 10 min. The resulting PCR products were sequenced and analyzed via BLAST (online tool, https://blast.ncbi.nlm.nih.gov/Blast.cgi, accessed on 25 March 2025), and phylogenetic analysis was performed using the Clustal algorithm and Neighbor-Joining method in MEGA 11.0 software [17]. The strain was deposited at the China General Microbiological Culture Collection Centre (CGMCC).

*Lactobacillus rhamnosus* GG ATCC 53103 (LGG), obtained from Yijia Biotechnology Co., Ltd. (Chengdu, China), served as the reference strain [18], owing to its well-established probiotic properties, characterized genetic features, and proven in vivo efficacy in modulating intestinal microbiota and enhancing gut barrier function [19].

### 2.2. Strain Safety Evaluation

Antibiotic susceptibility of HY0110 and LGG was evaluated using the Kirby–Bauer disk diffusion method. Bacterial glycerol stocks were revived by streaking onto MRS agar, and overnight cultures were centrifuged, washed, and standardized to an OD_600_ of 0.5 (approximately 1 × 10^8^ CFU/mL, verified by plate counting). These suspensions were uniformly spread on MRS agar plates, followed by the placement of antibiotic disks (ceftriaxone, tetracycline, gentamycin, clarithromycin, chloramphenicol, ampicillin, and clindamycin). After 18 h of incubation at 37 °C, the diameters of the inhibition zones were measured and interpreted in accordance with CLSI guidelines, categorizing the results as susceptible (S), intermediate (I), or resistant (R) [20].

### 2.3. Simulated Gastrointestinal Survival Assay

Gastrointestinal tolerance was assessed by sequentially subjecting bacterial strains to simulated gastric and intestinal environments. Overnight cultures (18 h, 37 °C) in MRS broth were centrifuged, washed, and standardized to an OD_600_ of 0.5 (approximately 1 × 10^8^ CFU/mL, confirmed by plate counting). These standardized suspensions were first exposed to simulated gastric fluid for 3 h at 37 °C, then transferred to simulated intestinal fluid for an additional 3 h. Viable cell counts were determined at the initial, post-gastric, and post-intestinal stages using standard plate counting methods. Survival rates were calculated as follows.

$$Survival\;rate\;(\%)=\frac{viable\;cell\;count\;after\;incubation}{viable\;cell\;count\;before\;incubation}\times 100\%,$$

LGG served as the reference strain, and all assays were performed in triplicate.

### 2.4. Antibacterial Assay

Antimicrobial activity was assessed using the Oxford cup method against *Escherichia coli* CMCC (B) 44102, *Salmonella* enterica serovar Paratyphi B CMCC (B) 50094, *Pseudomonas aeruginosa* CMCC (B) 10104, *Staphylococcus aureus* CMCC (B) 26003, and *Campylobacter jejuni* ATCC 33291. Bacterial strains were grown to the late logarithmic phase in Lysogeny Broth (LB), washed with PBS, and diluted to 10^8^ CFU/mL. LB agar plates (1.5% agar) were prepared with three equidistant Oxford cups. After 18 h of incubation at 37 °C, 200 μL of the cell-free supernatant (CFS) from strains HY0110 and LGG was added to the wells. Inhibition zones were measured, and statistical analysis was performed using Z-score evaluation [18].

### 2.5. Antioxidant Activity Assays

A suite of four in vitro assays was employed to evaluate antioxidant capacity. Hydroxyl radical scavenging was quantified via the Fenton reaction at 550 nm, whereas DPPH radical scavenging activity was measured at 517 nm, with results reported as micrograms of Trolox equivalents per milliliter. Total antioxidant capacity (T-AOC) was determined by monitoring Fe^3+^ reduction at 520 nm. Finally, superoxide anion scavenging was assessed using a xanthine–xanthine oxidase system, with vitamin C as a reference standard; the corresponding absorbance was recorded at 550 nm. All spectrophotometric measurements were made using 1 cm path length cuvettes.

### 2.6. Cancer Cell Cytotoxicity Analysis

#### 2.6.1. Cell Viability Assessment

The HT-29 cell line was kindly provided by Prof. Fangdong Zou (Key Laboratory of Bio-Resources and Eco-Environment, Ministry of Education, College of Life Sciences, Sichuan University, Chengdu, China). Cell viability was determined using the CCK-8 assay, which measures cellular metabolic activity based on the reduction of WST-8 to a colored formazan product. HT-29 cells (3000–5000 cells/well) were seeded in 96-well plates and incubated overnight at 37 °C in a humidified atmosphere containing 5% CO_2_. The next day, cells were treated with HY0110 supernatant at indicated concentrations or vehicle control for 24–48 h. Subsequently, 10 µL of CCK-8 solution was added to each well, and plates were incubated for an additional 1–2 h at 37 °C. Absorbance was measured at 450 nm using a microplate reader. Cell viability was calculated as a percentage relative to untreated control cells.

#### 2.6.2. Colony Formation Analysis with HY0110 Supernatant

For colony formation assays, HT-29 cells (2000 cells/well) were seeded in 6-well plates and cultured for 20 days in medium supplemented with 1% (*v*/*v*) HY0110 cell-free supernatant, which was refreshed every 48 h. Following incubation, cells were gently washed with PBS, fixed with 4% paraformaldehyde for 15–20 min, and stained with 0.5% crystal violet for approximately 30 min. Colonies were counted manually after rinsing and drying, and the average colony numbers ± SD from three independent experiments were recorded.

#### 2.6.3. Pro-Apoptotic Effects Analysis

To assess pro-apoptotic effects, HT-29 cells (2000 cells/well) seeded in 96-well plates were exposed to 4% HY0110 supernatant for 48 h. Cells were subsequently washed with PBS and lysed in ice-cold RIPA buffer (1% NP-40, 150 mM NaCl, 0.5% sodium deoxycholate, 0.1% SDS, and 50 mM Tris-HCl, pH 7.4) supplemented with protease inhibitors. After incubation on ice for 30 min, lysates were centrifuged at 12,000× *g* for 15 min at 4 °C. Protein concentrations in the clarified supernatants were quantified using the BCA assay. Lysates were mixed with 2× SDS sample buffer, denatured at 95 °C for 5 min, and stored at −20 °C until further analysis by Western blot. All samples were analyzed within two weeks to ensure protein integrity.

#### 2.6.4. Apoptosis Detection by One-Step TUNEL Assay

HT-29 cells treated with 4% HY0110 supernatant were fixed in 4% paraformaldehyde for 15 min and permeabilized with 0.1% Triton X-100 (Beijing Solarbio Science & Technology Co., Ltd., Beijing, China) in PBS on ice for 5 min. Apoptotic DNA fragmentation was assessed using a one-step TUNEL (Terminal deoxynucleotidyl transferase dUTP nick end labeling) assay according to the manufacturer’s instructions. This method labels 3′-OH DNA ends with fluorescently tagged dUTP via terminal deoxynucleotidyl transferase (TdT). Fluorescent microscopy was used to visualize apoptotic cells.

### 2.7. Animals and Treatments

#### 2.7.1. Animal Studies and Ethics Authorization

All procedures were reviewed and approved by the Animal Ethics Committee in the College of Life Sciences at Sichuan University (Protocol ID: SCU240412001).

Forty-five male ICR mice (6–7 weeks old, 20 ± 2 g) were procured from Chengdu Dashuo Experimental Animal Co. (Sichuan, China) and housed under specific pathogen-free (SPF) conditions at 20 ± 2 °C, 45 ± 10% humidity, on a 12 h light/dark cycle. After a one-week acclimatization with free access to food and water, mice were randomly allocated into three groups (n = 15 per group) and housed at five animals per cage throughout the experiment.

*E. faecalis* HY0110 was cultured in MRS broth at 37 °C for 24 h, yielding 10^8^ CFU/mL as confirmed by plate counts. For 14 consecutive days, the control group (CK) received a daily oral dose of sterile culture medium (0.2 mL/10 g body weight). The DSS group received the same sterile medium for seven days, and then 4% dextran sulfate sodium (DSS) solution for the subsequent seven days at the same dose volume. The prophylactic intervention group (HY0110) received a daily oral dose of the HY0110 suspension (10^8^ CFU/mL, 0.2 mL/10 g body weight) for seven days, followed by DSS administration under identical conditions.

#### 2.7.2. Inflammatory Cytokine Detection in Mouse Serum

Blood samples were collected via retro-orbital sinus puncture from strategically selected mice (one per cage, n = 3 per group) to optimize statistical validity while adhering to ethical 3Rs principles for minimizing invasive procedures in animal experimentation. Serum was harvested by centrifuging at 3000 rpm for 10 min. The concentrations of IL-1β, TNF-α, IL-6, and IL-8 were measured using commercial ELISA kits (Jiangsu Jingmei Biological Technology Co., Ltd., Yancheng, China) in accordance with the manufacturer’s instructions.

#### 2.7.3. Analysis of Changes in Mouse Gut Microbiota

Fecal samples (two per cage, n = 6 per group) were collected aseptically from each group following DSS treatment and stored at −80 °C for downstream analysis. Total genomic DNA was extracted using a CTAB/SDS protocol, and the V3–V4 hypervariable regions of the 16S rRNA gene were amplified using the 338F/806R primers. Sequencing was conducted on an Illumina NovaSeq PE250 platform (Illumina, San Diego, CA, USA) at Nuohe Zhiyuan Technology Co., Ltd. (Beijing, China), and raw data were deposited in the NCBI Sequence Read Archive (BioProject PRJNA1207272).

Taxonomic classification was performed in QIIME2 using the RDP classifier (v2.14) at a 97% identity threshold against the Silva138/16S reference database [21]. Microbial composition and differential abundance at both phylum and genus levels were analyzed via LEfSe [22]. Beta diversity was visualized by Principal Coordinates Analysis (PCoA) in QIIME2, with final plots generated in R using the ggplot2 and vegan packages. Functional gene prediction was carried out using PICRUSt with the COG database [23].

### 2.8. GC-MS Evaluation of Acid Output Capability

Fermentation metabolites produced by HY0110 were characterized by gas chromatography–mass spectrometry (GC–MS) using a Shimadzu GCMS-QP2010 Plus system (Shimadzu Corporation, Kyoto, Japan) equipped with an Rtx-5 capillary column (250 μm ID, 250 m length). The oven temperature program began at 40 °C, ramped to 150 °C at 5 °C/min, then to 280 °C at 10 °C/min, and was held at 280 °C for 2 min. Helium (99.999%) was used as the carrier gas at 1.0 mL/min. The interface and ion source were maintained at 220 °C and 200 °C, respectively, scanning the *m*/*z* range of 33–500.

For sample preparation, 2 mL of the fermented matrix was spiked with 200 μg/mL 2-methylbutyric acid as an internal standard. The injection was performed in split mode (1:3) at 270 °C with a 0.1 min solvent delay. Quantifications of acetic, butyric, and valeric acids were obtained, and antimicrobial activity was assessed using Mantel tests. Pearson correlation coefficients were calculated between short-chain fatty acid (SCFA) concentrations and the growth inhibition of five pathogenic strains [24].

### 2.9. Metabolomic Analysis of HY0110 Fermented P. americana Powder

#### 2.9.1. Sample Preparation

*E. faecalis* HY0110 was cultured at 37 °C for 96 h in sterile medium containing 10 g of *P. americana* powder, 3 g of oligofructose, and 100 mL of water. After fermentation, the culture was centrifuged at 4000 rpm for 10 min at 4 °C, and the resulting supernatant was filtered through a 0.22 μm membrane. Samples were prepared in triplicate, with an uninoculated medium serving as the control.

#### 2.9.2. LC-MS Analysis

Non-targeted metabolomic profiling was conducted at Nuohe Zhiyuan Technology Co., Ltd. (Beijing, China) utilizing a Q Exactive™ HF/Q Exactive™ HF-X mass spectrometer (Thermo Fisher, Niedersachsen, Germany) coupled with a Vanquish UHPLC system (Thermo Fisher, Niedersachsen, Germany). Chromatographic separation was performed on a Hypersil Gold column (C18, 100 × 2.1 mm) (Thermo Fisher, Niedersachsen, Germany) maintained at 40 °C with a flow rate of 0.2 mL/min. The mobile phases consisted of 0.1% formic acid in water (A) and methanol (B). The gradient elution program was: 0–1.5 min, 98% A and 2% B; 3 min, 15% A and 85% B; 10 min, 0% A and 100% B; 10.1–12 min, 98% A and 2% B for re-equilibration. Mass spectrometry was performed with an *m*/*z* range of 100–1500 in both positive and negative ESI modes. ESI source parameters were: spray voltage 3.5 kV, sheath gas flow rate 35 psi, auxiliary gas flow rate 10 L/min, capillary temperature 320 °C, S-lens RF level 60, and auxiliary gas heater temperature 350 °C. MS/MS fragmentation was conducted using data-dependent scans.

#### 2.9.3. Data Processing and Analysis

Raw data were processed in CD3.3 for peak detection, alignment, and normalization. Molecular features were putatively identified by matching against the HRMS, mzCloud, mzVault, and MassList databases (mass error < 10 ppm; MS/MS fragment score > 80%). Peak areas were normalized to the total ion current to enable relative quantification. Significant metabolites were filtered by VIP > 1, *p* < 0.05, and a fold-change threshold between fermented and non-fermented samples.

#### 2.9.4. Statistical and Bioinformatic Analysis

Differential metabolites were identified using Student’s *t*-test (*p* < 0.05) and a fold-change ≥ 2. Principal component analysis (PCA) was conducted in R (v4.4.2). Metabolic pathway enrichment was performed using the clusterProfiler package (v4.0) in R (v4.4.2) with KEGG annotations, with the enrichment factor defined as the ratio of differential metabolites to total metabolites per pathway [25].

### 2.10. Statistical Analysis

All statistical analyses were performed in R (v4.4.2). Data from at least three independent replicates are presented as mean ± standard deviation. Intergroup differences were evaluated by one-way ANOVA with Tukey’s post hoc test, and *p* < 0.05 was considered statistically significant.

## 3. Results and Discussion

### 3.1. Taxonomic Characterization of E. faecalis HY0110

**Figure 1:**
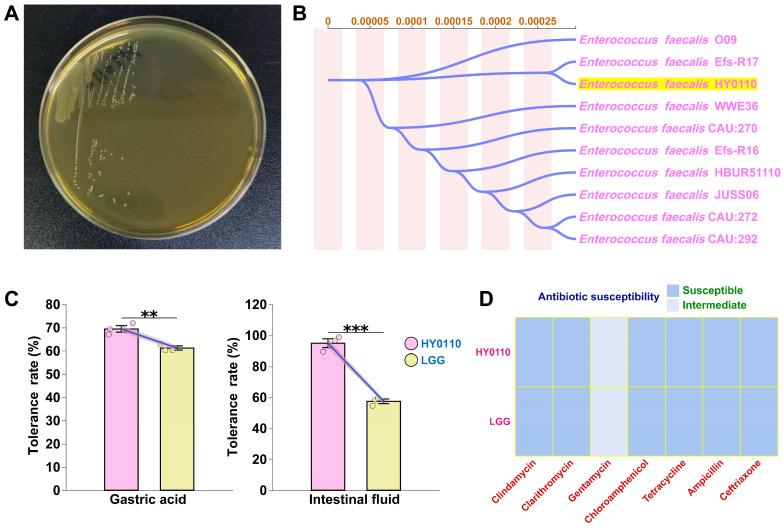
Morphology, identification, and safety assessment of the HY0110 strain. Notes: (**A**) Morphology of the HY0110 strain. (**B**) Phylogenetic tree of the HY0110 strain constructed using the NJ method. (**C**) Survival rate of the HY0110 strain in simulated gastrointestinal fluids (** *p* < 0.01, *** *p* < 0.001). (**D**) Antibiotic sensitivity test of the HY0110 strain against common antibiotics.

Sequence analysis of the complete 16S rRNA gene confirmed that strain HY0110 belongs to *E. faecalis* and shares 99.80% sequence identity with *E. faecalis* Efs-R17, suggesting a phenotypic traits characteristic of the *E. faecalis* clade. A phylogenetic tree generated using the Neighbor-Joining method further demonstrated the phylogenetic relationship between HY0110 and its closely related strains (Figure 1A,B).

### 3.2. Antibiotic Susceptibility Tests

Antibiotic resistance is a major threat in clinical and public health arenas, largely due to the risk of horizontal gene transfer under selective pressure [26]. Against this backdrop, we assessed HY0110’s antibiotic susceptibility profile as an essential safety parameter for food-grade strains. HY0110 exhibited high levels of susceptibility (S)—manifested by inhibition zones consistently greater than 15 mm—to the antibiotics tested (ceftriaxone, tetracycline, clarithromycin, chloramphenicol, ampicillin, and clindamycin). Notably, these findings aligned with the susceptibility pattern of LGG, indicating a similarly favorable risk profile (Figure 1D).

Such phenotypic susceptibility strongly suggested that HY0110 likely lacked major resistance determinants typically encoded on plasmids or within chromosomal segments prone to horizontal gene transfer. Although whole-genome sequencing would provide more definitive confirmation of the absence of genes such as *tet*(*M*) and *erm*(*B*) [27], our results pointed to a minimal likelihood of HY0110 contributing to the resistome in human populations: a conclusion that resonated with European Food Safety Authority (EFSA) and Food and Drug Administration (FDA) guidelines mandating robust antibiotic susceptibility data for probiotic strains [28]. Mechanistically, broad susceptibility often reflected limited prior exposure to antibiotic-rich environments, thereby precluding the emergence of adaptive features such as efflux pumps or modified target sites [29].

### 3.3. Gastrointestinal Transit Tolerance

HY0110 demonstrated exceptional resilience under simulated gastrointestinal conditions, a fundamental attribute for probiotic functionality. In gastric simulation, HY0110 maintained 69.4 ± 2.45% viability, significantly surpassing the reference strain LGG (61.2 ± 1.60%). More remarkably, during subsequent intestinal fluid exposure, HY0110 preserved 94.9 ± 4.88% viability, while LGG exhibited substantial decline to 57.5 ± 2.56%. Statistical analysis confirmed that HY0110 exhibited significantly higher tolerance than LGG in both gastric and intestinal environments (*p* < 0.01, Figure 1C), with final viable counts remaining above the critical threshold of 1 × 10^7^ CFU/mL required for probiotic efficacy.

The superior acid tolerance of HY0110 likely arises from multiple adaptive mechanisms evolved by the strain to withstand extreme pH fluctuations, including proton pumps, arginine deiminase systems, and membrane modifications maintaining cytoplasmic pH homeostasis [30]. Its notably high intestinal fluid tolerance further suggests effective bile salt hydrolase (BSH) activity, a key adaptation that deconjugates bile acids and enhances colonization capacity [31].

### 3.4. Antimicrobial, Antioxidant, and Antiproliferative Properties of Probiotic Strain HY0110

**Figure 2:**
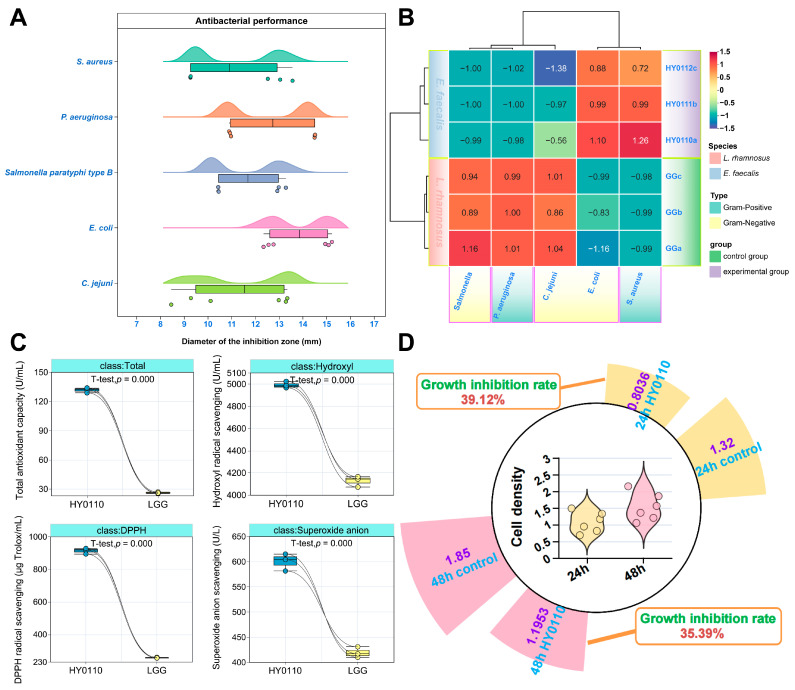
Exploration of the multifunctional profile of HY0110. Notes: (**A**,**B**) Inhibitory effects of HY0110 and LGG against common pathogenic bacteria. (**C**) Antioxidant activity assessment of HY0110 and LGG. (**D**) Inhibitory effects of HY0110 on HT-29 cells.

Strain HY0110 exhibited potent antimicrobial, antioxidant, and antiproliferative activities, in line with established probiotic mechanisms involving bacteriocins, organic acids, and antioxidant enzymes [18,32,33]. Compared to LGG, HY0110 showed stronger antagonism against five zoonotic pathogens, particularly *E. coli*, as demonstrated by larger inhibition zones (11.75 mm vs. 10.80 mm, Figure 2A,B). This heightened efficacy may be linked to elevated bacteriocin or organic acid production [18], paralleling findings in other well-characterized probiotic strains [34]. Such attributes suggest that HY0110 can reduce pathogenic load and enhance food safety.

HY0110 also demonstrated significantly greater antioxidant activity than LGG (*p* < 0.001, Figure 2C), with total antioxidant capacity outstripping that of LGG by approximately fivefold. Comparable improvements in DPPH and hydroxyl radical scavenging were observed, presumably stemming from specialized metabolic pathways involving metal ion chelation and exopolysaccharide biosynthesis [35]. These antioxidant properties may be crucial in preventing oxidative stress, supporting gut health, and optimizing nutrient utilization, further highlighting HY0110’s suitability for functional food applications.

In addition, HY0110 fermentation supernatant reduced HT-29 colorectal cancer cell proliferation by around 35–40% (Figure 2D), suggesting a role in modulating epigenetic and oncogenic pathways [36]. Incorporating HY0110 into functional food formulations may, thus, offer multifaceted health benefits, including immune support and enhanced intestinal homeostasis.

Overall, compared to LGG, HY0110 demonstrates marked advantages in gastrointestinal tolerance, antimicrobial efficacy, antioxidant capacity, and tumor cell proliferation inhibition. From an application standpoint, its robust antioxidant and anticancer potential, coupled with high survivability, offers a broader and more precisely targeted functional scope in both functional foods and adjuvant therapies, particularly for populations at heightened risk of gastrointestinal disorders or oxidative stress. Moreover, the strain’s marine origin implies an expanded genetic repertoire for metabolic pathways and adaptive strategies, providing valuable avenues for future strain optimization and synthetic biology-based enhancements.

### 3.5. Effect of HY0110 Strain on Sodium Polysaccharide-Induced Colitis in Mice

In vivo validation is crucial for identifying probiotics with tangible health benefits in functional foods [37]. A lactic acid bacterium derived from *T*. *thynnus* was shown to mitigate inflammation and gut dysbiosis in a DSS-induced colitis model, suggesting its promise as a probiotic for improving intestinal well-being. Notably, this strain lowered pro-inflammatory cytokines and altered gut microbial composition and diversity, consistent with anti-inflammatory effects. Such findings endorse its potential to enhance digestive health, an essential facet of overall nutritional performance.

#### 3.5.1. Anti-Inflammatory Effects of HY0110 in DSS-Induced Colitis Model

Administering DSS prompted a marked elevation in pro-inflammatory cytokines (IL-1β, IL-6, IL-8, TNF-α; *p* < 0.05, Figure S1). Crucially, HY0110 supplementation restored IL-1β and IL-6 to control values (*p* < 0.05 vs. DSS, Figure S1A,B), demonstrating potent anti-inflammatory activity. Given IL-1β’s role in immune cell recruitment and IL-6’s amplification of pro-inflammatory cascades [38], normalizing these factors implicates the modulation of the IL-1β/IL-6 axis as a key mechanism of HY0110’s therapeutic effect, aligning with established probiotic actions in colitis models.

#### 3.5.2. Examination of the Intestinal Microbiome Across Different Mouse Cohorts

**Figure 3:**
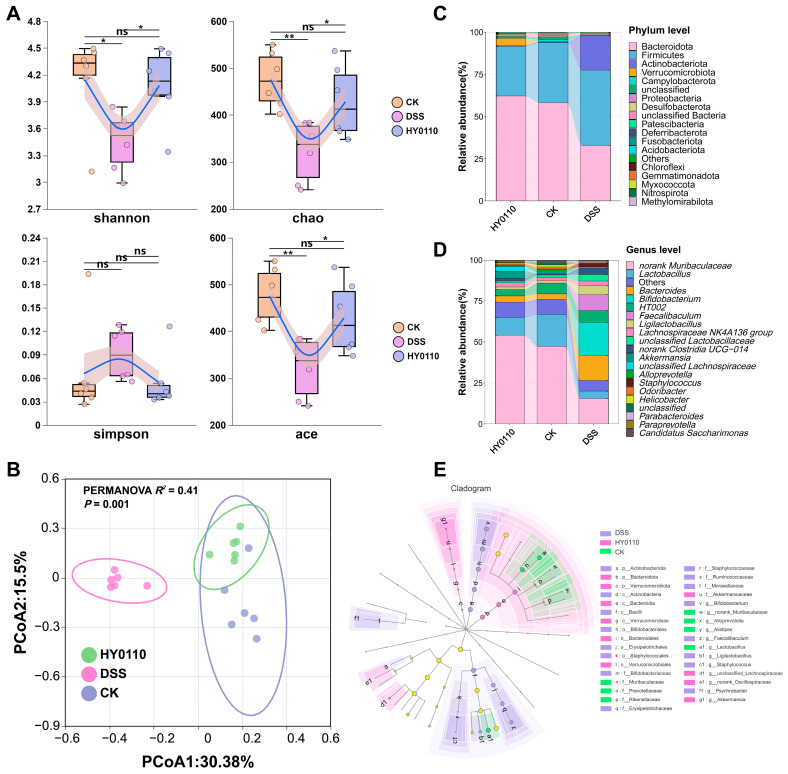
Analysis of gut microbiota structural changes in DSS and HY0110 intervention mice. Notes: (**A**,**B**) Alpha diversity (shannon, chao1, simpson, ace) and beta diversity (PCoA) changes in gut OTU microbiota across groups (* *p* < 0.05, ** *p* < 0.01, ns = not significant). (**C**,**D**) Changes in gut bacterial phylum- and genus-level composition across groups. (**E**) LEfSE analysis of gut microbiota across groups.

The analysis of microbial community structure using OTU abundance revealed significant alterations in both alpha diversity (local species richness and evenness) and beta diversity (compositional differences among communities) [39]. DSS administration led to a notable decrease in microbial richness and diversity, as reflected by reductions in the ACE, Chao, and Shannon indices (*p* < 0.05). Intervention with HY0110 restored these indices to levels comparable to the control group (Figure 3A). PCoA (PERMANOVA: *R*^2^ = 0.41, *p* = 0.001, Figure 3B) showed distinct clustering patterns among the groups, with HY0110-treated mice clustering closely with controls and the DSS group displaying clear separation.

In terms of taxonomy, DSS exposure reduced Bacteroidota (from 60.25 ± 8.75% to 32.93 ± 8.14%) and increased Firmicutes (from 32.58 ± 8.86% to 44.61 ± 14.30%; Figure 3C), alongside elevated potentially pathogenic Actinobacteriota and Staphylococcaceae [40]. HY0110 supplementation counteracted these dysbiotic patterns, restoring beneficial genera such as *Lactobacillus* and *Bacteroides* (Figure 3D), known to produce SCFAs and support gut barrier integrity [41]. LEfSe analysis showed additional enrichment of *Akkermansia* and a return of the protective Muribaculaceae family (Figure 3E) [42]. These observations echo dysbiosis profiles in IBD patients and suggest that HY0110 alleviates colitis by reestablishing microbial equilibrium and suppressing pathogenic taxa [43].

**Figure 4:**
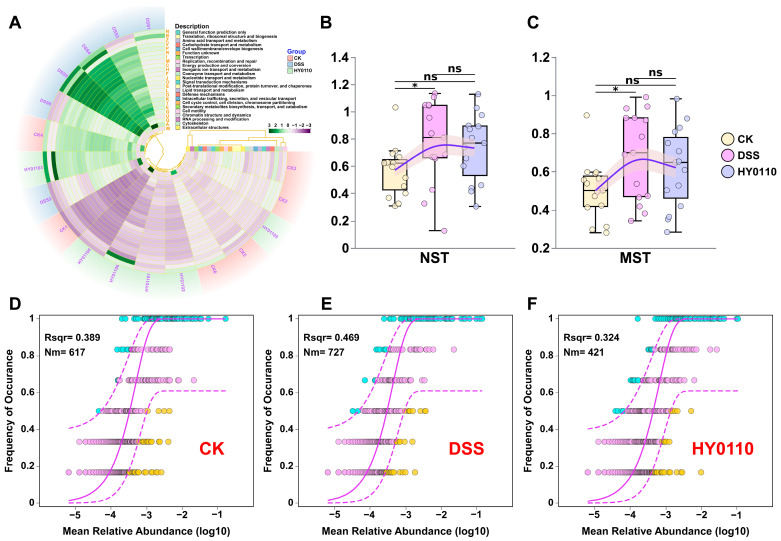
Exploration of gut microbiota function and assembly mechanisms in mice. Notes: (**A**) COG-based functional prediction of gut microbiota changes across groups. (**B**,**C**) Assessment of NST in gut microbiota structure across groups using normalized (**B**) and modified (**C**) stochasticity tests (* *p* < 0.05, ns = not significant). (**D**–**F**) NCM analysis of gut microbiota structure across groups.

PICRUSt analysis indicated substantial changes in microbial metabolic functions in DSS-induced colitis, with partial recovery following HY0110 treatment (Figure 4A). This functional restoration suggests that mitigating colitis involves stabilizing key microbial functions, such as nutrient absorption, vitamin synthesis, and the metabolism of bile acids and xenobiotics [44].

Microbial community assembly is driven by two complementary processes: deterministic niche-based selection and stochastic events such as ecological drift. In the DSS-induced colitis model, applying the Neutral Community Model (NCM) in conjunction with the Normalized Stochasticity Ratio (NST) [45,46] revealed that DSS treatment significantly increased stochastic influences—evidenced by higher NST values (*p* < 0.05, Figure 4B,C), as well as elevated Nm (727 vs. 617 and 421) and Rsqr (0.469 vs. 0.389 and 0.324, Figure 4D–F)—ultimately destabilizing the gut microbiota. These results support the ecological theory suggesting that environmental stress fosters stochastic rather than deterministic community assembly [47]. In contrast, HY0110 supplementation mitigated these disruptions, shifting the community assembly mechanism toward a more deterministic regime similar to that of healthy controls. This transition promotes the ecological filtering and niche-based selection of beneficial taxa, yielding more resilient microbial communities. From a therapeutic standpoint, such approaches can precisely sculpt microbial configurations, offering a mechanistically substantiated strategy for managing chronic inflammatory bowel conditions.

#### 3.5.3. Comparison with Existing Probiotic Strategies for Gut Microbiota and Immunomodulation

In a DSS-induced colitis mouse model, supplementation with single-strain HY0110 effectively restored gut microbial diversity, re-enriched beneficial taxa, and suppressed potentially pathogenic bacteria, while downregulating pro-inflammatory cytokines such as IL-1β and IL-6. This multifaceted protection of the intestinal mucosal barrier and immune homeostasis aligns with previous probiotic intervention findings [48]. For instance, *Lactobacillus plantarum* A3, isolated from equine feces, similarly reduced disease severity and modulated cytokine profiles in DSS-treated mice [49]; iron-enriched *Lactobacillus alimentarius* NKU556-Fe enhanced iron absorption while alleviating colonic inflammation [50]; and multi-strain formulations (e.g., *Lactobacillus reuteri*, *Bacillus coagulans*, *Bifidobacterium longum*, *Clostridium butyricum*) demonstrated synergistic anti-inflammatory and mucosal repair benefits surpassing those of single-strain or conventional treatments [51]. Compared with these diverse probiotic approaches, HY0110 alone produced comparable microbiota remodeling and immunomodulatory efficacy, underscoring its strong mucosal protection and anti-inflammatory potential. Moreover, various probiotic strains or combinations confer distinct advantages—such as SCFA production or iron enrichment—implying that HY0110’s dual support for microbial homeostasis and immune regulation could be leveraged independently or integrated into optimized multi-strain regimens for future IBD management.

### 3.6. Major Antibacterial Components–Acid-Producing Capacity and Constituent Analysis

**Figure 5:**
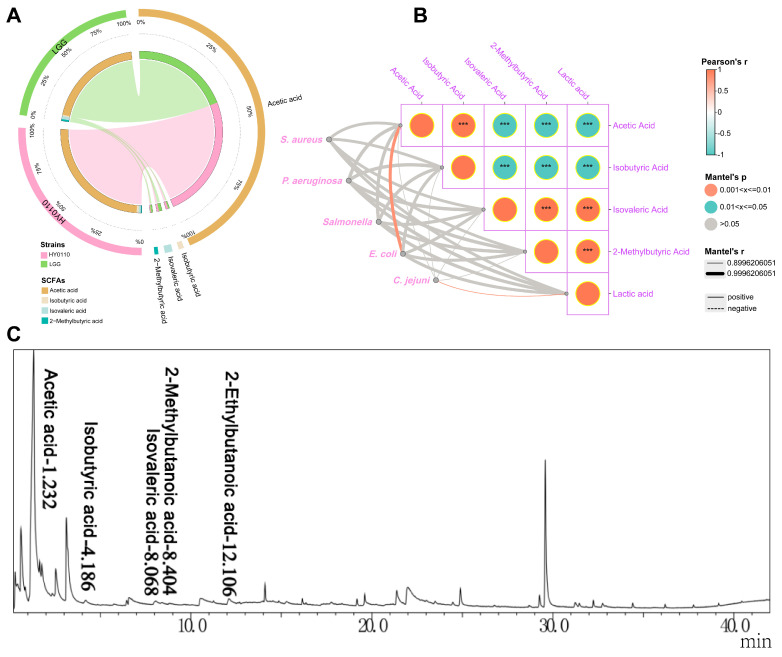
GC-MS examination of SCFA production by HY0110 and LGG strains. Notes: (**A**) The concentration of SCFAs produced by each strain. (**B**) The relationship between the inhibitory activity of each strain against specific pathogens and their SCFA levels. *** *p* < 0.001. (**C**) The chromatogram illustrates the ion flow for quantifying SCFAs in HY0110 through gas GC-MS.

The HY0110 strain produced notably higher levels of acetic acid (16.505 μg/mL) compared to LGG (13.08 μg/mL), alongside a distinctive short-chain fatty acid (SCFA) profile characterized by varying concentrations of branched-chain fatty acids, such as isobutyric (0.288 μg/mL vs. 0.18 μg/mL), isovaleric (0.283 μg/mL vs. 0.41 μg/mL), and 2-methylbutyric acids (0.115 μg/mL vs. 0.23 μg/mL), as revealed by GC-MS (Figure 5A). High-resolution chromatographic separation with minimal peak interference validated HY0110’s enhanced fermentative capacity relative to LGG, facilitating precise metabolite quantification (Figure 5C). Lactic and acetic acids, key organic acids produced by lactic acid bacteria (LAB), underlie crucial antimicrobial mechanisms [18]. Concurrently, the biosynthesis of fatty acids, bioactive peptides, and reactive oxygen species further contributes to pathogen inhibition and gut microbiota modulation, ultimately supporting host health [52].

SCFAs, which are the predominant metabolites arising from bacterial fermentation in the intestine, play critical roles in maintaining epithelial integrity, modulating the immune system, exerting protective effects against tumors, and regulating bacterial populations [53]. In this study, acetic acid emerged as the principal SCFA and serves as a precursor for both cholesterol and butyric acid synthesis, as well as a vital component of bacterial fermentation [54]. Correlation analyses revealed a strong positive association (R ≥ 0.999; *p* < 0.01) between acetic acid levels and antibacterial activity against *E. coli*, underscoring its potential for enhancing food safety and curbing pathogen proliferation (Figure 5B). Mechanistically, acetate has been shown to dissipate intracellular pH gradients, force bacterial cells to expend additional energy on proton extrusion, and promote the accumulation of toxic intermediates, collectively leading to impaired growth and viability in *E. coli* [55]. Concurrently, other SCFAs (e.g., isobutyric and isovaleric acids) may exert synergistic or complementary inhibitory effects, possibly by altering membrane permeability or interfering with nutrient transport. Such multifactorial modes of action align with known probiotic strategies, wherein organic acids, bacteriocins, and reactive metabolites collectively reinforce the antimicrobial spectrum [18].

Beyond its in vitro pathogen-suppressive effects, the elevated acetic acid output of HY0110 also has functional implications for gut health. Acetate serves as both a microbial fermentative end-product and a key metabolic substrate for host cells, contributing to cholesterol and butyric acid biosynthesis [56]. Consequently, HY0110’s robust SCFA production could influence host lipid metabolism and strengthen gut barrier integrity. Moreover, SCFA generation has been linked to immune modulation, epithelial cell regulation, and support of beneficial microbiota [57], underscoring the ecological value of introducing HY0110 into the gastrointestinal environment. Future research focusing on the gene-level regulation of acid production—combined with in vivo validation—would further clarify how HY0110 orchestrates these antimicrobial mechanisms, informing refined food safety strategies and therapeutic interventions aimed at maintaining gastrointestinal homeostasis.

### 3.7. Mechanism of HT-29 Cell Inhibition

**Figure 6:**
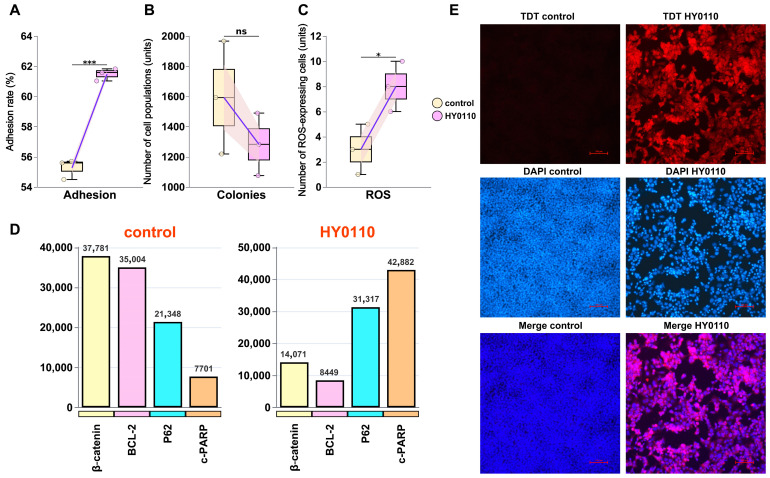
Exploration of the mechanism by which HY0110 inhibits HT-29 cells. Notes: (**A**) Adhesion rates of HY0110 and LGG to HT-29 cells (*** *p* < 0.001). (**B**) Effect of HY0110 fermentation supernatant on HT-29 colony formation (ns = not significant). (**C**) Intracellular ROS levels in HT-29 cells after HY0110 treatment (* *p* < 0.05). (**D**) Regulation of apoptotic protein expression by HY0110 in HT-29 cells. (**E**) TUNEL assay showing apoptosis in HT-29 cells after HY0110 treatment.

The novel probiotic HY0110 exhibits multifaceted bioactive properties that hold considerable promise for advancing functional food innovation and improving consumer health. It demonstrates pronounced anti-tumor effects through mechanisms involving both cellular viability and immune pathways. Compared to the well-known probiotic LGG, HY0110 displayed significantly higher adhesion rates (61.48 ± 0.41%; *p* < 0.05, Figure 6A), indicating a strong capacity for intestinal colonization: a crucial factor in maintaining gut health and optimizing nutrient absorption. This adhesion likely involves specific interactions between bacterial surface molecules (e.g., mucin- and fibronectin-binding proteins) and corresponding epithelial receptors [58]. Vergalito et al. demonstrated that highly adherent probiotics can occupy binding sites otherwise exploited by pathogenic bacteria that drive inflammation and DNA damage [59]. Moreover, exopolysaccharides produced by the probiotic may reinforce this protective effect by forming biofilm-like structures, physically shielding epithelial cells from toxin exposure.

In HT-29 cells, HY0110 supernatant treatment reduced colony formation to 80.54% of control levels (1283 ± 207 vs. 1593 ± 374 colonies; Figure 6B) while concurrently increasing intracellular reactive oxygen species (ROS; Figure 6C). These heightened ROS levels likely trigger oxidative-stress-dependent apoptosis through mitochondrial membrane permeabilization, cytochrome c release, and subsequent caspase activation [60], consistent with Gholipour et al., who reported comparable cytotoxic mechanisms in HT-29 cells exposed to probiotic metabolites [61]. Western blot analyses further revealed the downregulation of β-catenin (14,071) and BCL-2 (8449) alongside the upregulation of P62 (31,317) and c-PARP (42,882), indicative of both intrinsic and extrinsic apoptotic pathways (Figure 6D). This suppression likely involves GSK-3β-mediated β-catenin phosphorylation leading to proteasomal degradation and the disruption of Wnt signaling [62], while diminished BCL-2 levels shift the BAX/BCL-2 ratio toward pro-apoptotic signaling, compromising mitochondrial integrity [63]. Singh et al. likewise demonstrated that probiotic-derived SCFAs modulate these pathways by inhibiting histone deacetylases, thereby epigenetically steering cancer cells toward apoptosis [64]. TUNEL assays confirmed a more than sevenfold elevation in fluorescence intensity (287 ± 42 vs. 38 ± 4), signifying enhanced apoptosis (Figure 6E).

Such molecular and cellular mechanisms bear significant clinical implications. Epidemiological evidence has linked probiotic intake to reduced colorectal cancer incidence [65], and clinical trials suggest that certain strains lower cancer biomarkers in high-risk populations [66]. By integrating direct cytotoxicity, apoptosis induction, microbiome modulation, and immune system regulation, HY0110 addresses multiple cancer hallmarks simultaneously. This multi-targeted strategy, thus, positions HY0110 as a promising candidate for colorectal cancer prevention and adjunct therapy.

### 3.8. Metabolic Profiling of HY0110 Fermented and Non-Fermented P. americana Powder

#### 3.8.1. HY0110 Fermentation Improves Bioavailability and Therapeutic Potential of *P. americana* Powder

Fermenting *P. americana* powder with HY0110 substantially boosts its bioavailability and bioactivity through microbial biotransformation, which diminishes harmful components and enriches beneficial metabolites, including organic acids and bioactive peptides. This dual effect not only improves the safety profile of insect-derived ingredients, but also elevates their nutritional and functional potential, aligning with efforts to develop more efficacious functional foods. In light of these findings, HY0110-fermented *P. americana* powder exhibits considerable potential for fortifying immune function and improving gut health, thereby offering a novel nutrient-rich strategy to address the evolving demands for advanced functional foods.

#### 3.8.2. PCA Reveals Metabolic Differentiation

**Figure 7:**
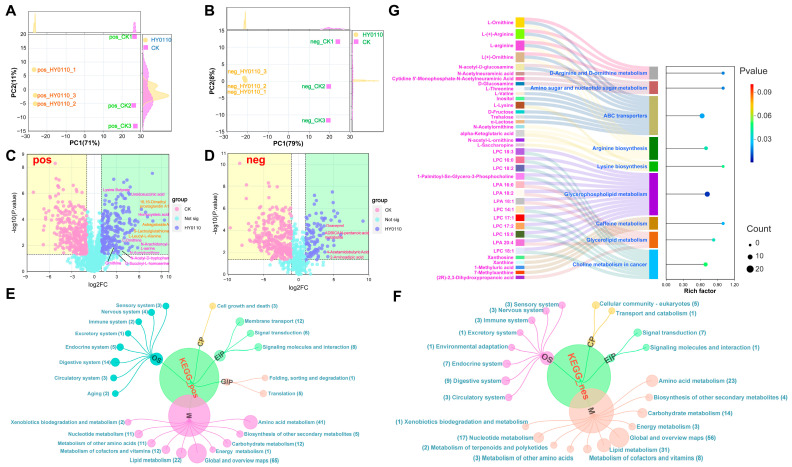
Metabolic profiling and pathway enrichment analysis of HY0110-fermented *P. americana* powder in both positive and negative ion modes. Notes: (**A**,**B**) PCA of HY0110-fermented and control samples in positive (**A**) and negative (**B**) ion modes. (**C**,**D**) Differential metabolite profiles of HY0110-fermented and control samples in positive (**C**) and negative (**D**) ion modes. (**E**,**F**) KEGG pathway enrichment analysis of metabolites in both positive (**E**) and negative (**F**) ion modes following HY0110 fermentation. (**G**) Significantly enriched KEGG pathways following HY0110 fermentation of *P. americana* powder.

The PCA demonstrated clear metabolic distinctions between HY0110-fermented and control samples in both ion modes. The first principal component (PC1) accounted for over 70% of the variance, and together PC1 and PC2 explained 87% (negative ion mode) and 82% (positive ion mode) of the total variation (Figure 7A,B). These pronounced metabolic shifts, coupled with high reproducibility across biological replicates, suggest a strong impact of HY0110 fermentation on the metabolic landscape.

#### 3.8.3. Significant Increases in Amino Acids and Peptides

After 96 h of HY0110 fermentation, the amino acid content of the *P. americana* supernatant rose significantly, over tenfold for compounds such as N-arachidonoyl-L-serine, succinyl-homoserine, alloisoleucine, and homocysteine compared to non-fermented controls. Peptide concentrations also surged, led by a 16.89-fold increase in L-Leucyl-L-Alanine and a 7.95-fold increase in S-lactoylglutathione. These biomolecules are critical for protein synthesis, immune regulation, and processes such as enzyme activation and membrane permeability [67]. Moreover, astragaloside A increased by 668.73-fold—offering anti-inflammatory, hepatoprotective, and antitumor benefits [68]—whereas 16,16-dimethyl prostaglandin A1 rose 116.15-fold (Figure 7C,D), conferring a wide array of bioactivities.

This substantial enrichment in bioactive metabolites underscores the value of HY0110-fermented *P. americana* powder as a nutritionally enhanced ingredient suitable for functional food formulations aimed at improving gut health, immune responsiveness, and overall well-being. Such fermentation-driven strategies highlight an innovative approach for enhancing both safety and efficacy in insect-based food products.

#### 3.8.4. KEGG Pathway Remodeling

KEGG pathway analysis revealed extensive metabolic restructuring, with 65 and 56 metabolites altered in positive and negative ion modes, respectively. Pathways related to amino acid metabolism (41 and 23 metabolites), membrane transport (12 metabolites, positive mode), digestive systems (14 metabolites, positive mode), lipid metabolism (31 metabolites, negative mode), and carbohydrate metabolism (14 metabolites, negative mode) were significantly affected (Figure 7E,F).

#### 3.8.5. Key Enriched Pathways

Nine pathways displayed significant enrichment (*p* < 0.05). Glycerophospholipid metabolism emerged as a prominent pathway, encompassing 12 differential metabolites, mostly lysophosphatidylcholines (LPCs) and lysophosphatidic acids (LPAs). Lysine biosynthesis and choline metabolism exhibited particularly high enrichment scores (rich factor > 0.9). Other pathways such as D-arginine/D-ornithine metabolism, ABC transporters, and caffeine metabolism also demonstrated noteworthy alterations (Figure 7G).

#### 3.8.6. Metabolic Reorganization and Commercialization Challenges of HY0110-Fermented *P. americana* Powder

Fermentation-driven metabolic changes in HY0110-fermented *P. americana* powder indicate significant biochemical reorganizations. Enhanced amino acid metabolism points to increased protein turnover and bioactive peptide production [69]. Shifts in lipid metabolism and membrane transport suggest improved bioavailability through heightened membrane permeability [70]. Additionally, the enrichment in glycerophospholipid metabolism may lead to alterations in membrane architecture and cell signaling, while lysine biosynthesis supports protein synthesis and tissue growth. Choline metabolism further underpins neurotransmitter production and lipid transport [71].

However, several challenges hinder the large-scale commercialization of HY0110-fermented *P. americana* powder. Standardizing fermentation parameters, such as inoculum concentration, duration, and environmental conditions, is essential for ensuring consistent metabolic profiles and product quality. Regulatory compliance remains a significant concern, given the novel status of insect-derived ingredients in many markets, necessitating rigorous safety assessments and precise labeling. Consumer acceptance poses another challenge, as cultural perspectives on insect-based products vary, making the effective communication of nutritional and functional benefits crucial. Furthermore, preserving metabolite stability and bioavailability during storage and distribution demands careful formulation strategies. Lastly, economic feasibility, from raw material sourcing to scale-up, must be thoroughly evaluated to ensure both affordability and profitability. Addressing these interconnected scientific, regulatory, and consumer-oriented challenges will determine the success of HY0110-fermented *P. americana* powder in the functional food sector.

## 4. Conclusions

The marine-derived probiotic *E. faecalis* HY0110, isolated from *T*. *thynnus*, demonstrated enhanced probiotic functionality compared to the standard probiotic LGG, as evidenced by multi-omics investigations and in vivo validation. It not only matched LGG in antibiotic susceptibility and tolerance to adverse gastrointestinal conditions, but also exhibited functionally superior antimicrobial and antioxidant activities—largely attributable to increased acetic acid production—highlighting its potential to lower pathogenic load and support gut health within functional food applications. HY0110’s antimicrobial and antioxidant effects surpassed those of LGG, and it exhibited strong antiproliferative activity against HT-29 colorectal cancer cells by attenuating β-catenin and BCL-2 expression while upregulating pro-apoptotic markers P62 and c-PARP.

In the DSS-induced colitis model, HY0110 effectively attenuated inflammatory responses, enhanced the abundance of beneficial genera such as *Lactobacillus* and *Akkermansia*, thereby restoring gut microbial equilibrium, and shifted microbial community assembly dynamics toward more deterministic processes, demonstrating its significant role in maintaining intestinal homeostasis. Additionally, the fermentation of *P. americana* powder by HY0110 induced significant metabolic remodeling, notably elevating beneficial metabolites, including astragaloside A, thereby enhancing the nutritional and functional quality of insect-derived food products.

Collectively, these findings underscore the pivotal role of marine-derived probiotics in advancing functional food research, boosting functional food applications and providing novel nutritional health strategies. Thus, this study established a transformative paradigm for utilizing marine probiotics and entomological substrates in functional foods, offering strategic pathways for precision nutrition and inflammatory disease management.

## Data Availability

The original contributions presented in the study are included in the article/Supplementary Material, further inquiries can be directed to the corresponding authors.

## Supplementary Materials

The following supporting information can be downloaded at: https://www.mdpi.com/article/10.3390/foods14071181/s1. The Supplementary Material S1: 16S rDNA sequence analysis for identification of strain HY0110. Figure S1: Effects of HY0110 on pro-inflammatory cytokine levels in DSS-induced inflammatory models. Different letters (a, b) indicate significant differences between groups (*p* < 0.05), while shared letters indicate no significant difference.

## Abbreviations

The following abbreviations are used in this manuscript: DSS: dextran sulfate sodium; ELISA: Enzyme-Linked Immunosorbent Assay; GC-MS: gas chromatography–mass spectrometry; IBD: inflammatory bowel disease; IL: Interleukin; KEGG: Kyoto Encyclopedia of Genes and Genomes; LGG: *Lactobacillus rhamnosus* GG; MRSA: Methicillin-resistant *Staphylococcus aureus*; NF-κB: Nuclear Factor kappa-light-chain-enhancer of activated B cells; PCA: principal component analysis; ROS: reactive oxygen species; SCFA: short-chain fatty acid; TNF-α: Tumor Necrosis Factor alpha; TUNEL: Terminal deoxynucleotidyl transferase dUTP nick end labeling.
