# Supplementary material for: Marine-Derived Enterococcus faecalis HY0110 as a Next-Generation Functional Food Probiotic: Comprehensive In Vitro and In Vivo Bioactivity Evaluation and Synergistic Fermentation of Periplaneta americana Extract Powder
Source: Foods. 2025 Mar 28;14(7):1181. doi: 10.3390/foods14071181 (PMC11988638; doi:10.3390/foods14071181)
Supplement: Supplementary file 1 [file foods-14-01181-s001.zip › Supplementary Material S1.pdf]

HY0110:

GGCGGTTGGCGGGTGCTATACATGCAGTCGACGCTTCTTTCCTCCCGAGTG
CTTGCACTCAATTGGAAAGAGGAGTGGCGGACGGGTGAGTAACACGTGGG
TAACCTACCCATCAGAGGGGGATAACACTTGGAAACAGGTGCTAATACCGC
ATAACAGTTTATGCCGCATGGCATAAGAGTGAAAGGCGCTTTCGGGTGTCG
CTGATGGATGGACCCGCGGTGCATTAGCTAGTTGGTGAGGTAACGGCTCAC
CAAGGCCACGATGCATAGCCGACCTGAGAGGGTGATCGGCCACACTGGGA
CTGAGACACGGCCCAGACTCCTACGGGAGGCAGCAGTAGGGAATCTTCGG
CAATGGACGAAAGTCTGACCGAGCAACGCCGCGTGAGTGAAGAAGGTTTT
CGGATCGTAAAACTCTGTTGTTAGAGAAGAACAAGGACGTTAGTAACTGA
ACGTCCCCTGACGGTATCTAACCAGAAAGCCACGGCTAACTACGTGCCAGC
AGCCGCGGTAATACGTAGGTGGCAAGCGTTGTCCGGATTTATTGGGCGTAA
AGCGAGCGCAGGCGGTTTCTTAAGTCTGATGTGAAAGCCCCCGGCTCAAC
CGGGGAGGGTCATTGGAAACTGGGAGACTTGAGTGCAGAAGAGGAGAGT
GGAATTCCATGTGTAGCGGTGAAATGCGTAGATATATGGAGGAACACCAGT
GGCGAAGGCGGCTCTCTGGTCTGTAACTGACGCTGAGGCTCGAAAGCGTG
GGAGCAAACAGGATTAGATACCCTGGTAGTCCACGCCGTAAACGATGAGT
GCTAAGTGTTGGAGGGTTTCCGCCCTTCAGTGCTGCAGCAAACGCATTAAG
CACTCCGCCTGGGGAGTACGACCGCAAGGTTGAAACTCAAAGGAATTGAC
GGGGGCCCGCACAAGCGGTGGAGCATGTGGTTTAATTCGAAGCAACGCGA
AGAACCCTTACCAGGTCTTGACATCCTTTGACCACTCTAGAG
