# Supplementary figures and images for: Marine-Derived Enterococcus faecalis HY0110 as a Next-Generation Functional Food Probiotic: Comprehensive In Vitro and In Vivo Bioactivity Evaluation and Synergistic Fermentation of Periplaneta americana Extract Powder
Source: Foods. 2025 Mar 28;14(7):1181. doi: 10.3390/foods14071181 (PMC11988638; doi:10.3390/foods14071181)

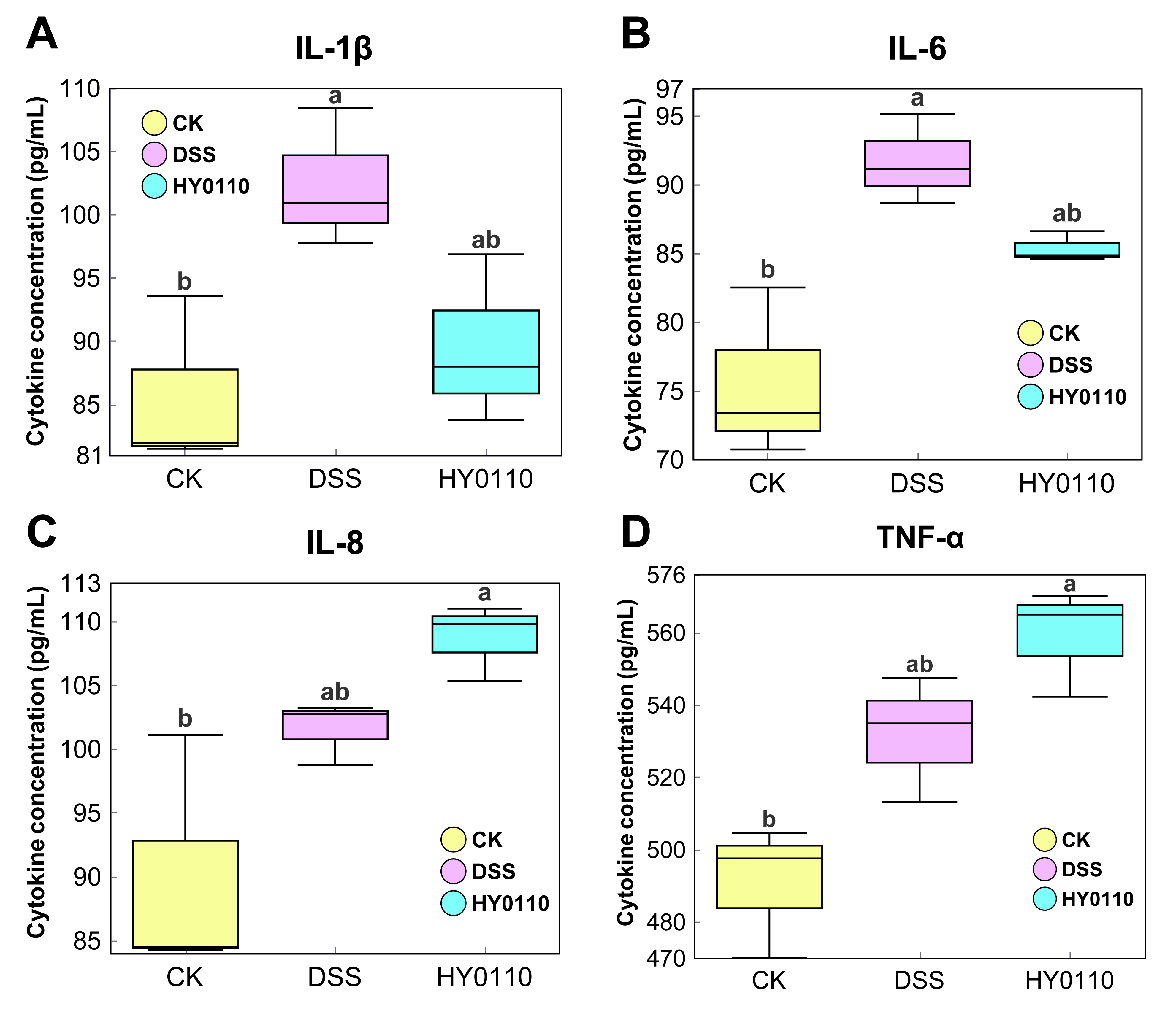

Supplement: Supplementary file 1 [file foods-14-01181-s001.zip › Figure S1.jpg]
